# Mechanisms and therapeutic potential of the hedgehog signaling pathway in cancer

**DOI:** 10.1038/s41420-025-02327-w

**Published:** 2025-02-03

**Authors:** Ge Cong, Xingyu Zhu, Xin Ru Chen, Hao Chen, Wei Chong

**Affiliations:** 1 Department of Gastrointestinal Surgery, Shandong Provincial Hospital Affiliated to Shandong First Medical University, 250021 Jinan, China; 2 Shandong Provincial Laboratory of Translational Medicine Engineering for Digestive Tumors, Shandong Provincial Hospital, 250021 Jinan, China; 3 Medical Science and Technology Innovation Center, Shandong First Medical University & Shandong Academy of Medical Sciences, 250021 Jinan, China; 4 Clinical Research Center of Shandong University, Clinical Epidemiology Unit, Qilu Hospital of Shandong University, 250021 Jinan, China

**Keywords:** Gastric cancer, Gastric cancer

## Abstract

A sort of major malignant disease, cancer can compromise human health wherever. Some mechanisms of the occurrence and evolution of cancer still seem elusive even now. Consequently, the therapeutic strategies for cancer must continually evolve. The hedgehog signaling pathway, a critical mediator in the normal development of numerous organs and the pathogenesis of cancer, is typically quiescent but is aberrantly activated in several malignancies. Extensive research has delineated that the aberrant activity of the hedgehog signaling pathway, whether autocrine or paracrine, is implicated in the initiation and progression of various neoplasms, including medulloblastoma (MB), basal cell carcinoma (BCC) and so on. Thus, notably Smo inhibitors, the opening of inhibitors of the hedgehog signaling pathway has become a topic of research attention. This review aims to summarize four aberrant activation pathways and the influence of hedgehog signaling pathway associated chemicals on tumor formation and development. Additionally, it will explore the therapeutic potential of targeted interventions in the hedgehog signaling pathway for cancer treatment.

## Facts

1. The overactivation or inhibition of Hedgehog pathways may be the key to triggering tumors.
2. The application of inducers and inhibitors is crucial in the study of the Hedgehog pathways and related cancers.
3. Targeting these interactions about the Hedgehog pathway interacts with other signaling networks in cancer may be a useful treatment strategy.

## Open questions

1. Whether and how to develop more efficient inducers and inhibitors of Hedgehog pathway?
2. What are the mechanisms of Hedgehog pathways during early carcinogenesis?
3. How might Hedgehog pathway inhibitors be made more effective for various cancer types?

## Introduction

The Hedgehog gene was found in Drosophila due to the remarkable phenotype of fly larvae lacking Hh. Mutant larvae fail to develop the segmented anterior-to-posterior body plan and have ectopic denticles resembling a hedgehog [1].Hedgehog (Hh) proteins constitute one family of a small number of secreted signaling proteins, the core components of which are the secreted molecule Hh, the twelve-pass transmembrane receptor Patched (PTCH), the seven-pass transmembrane co-receptor Smoothened (SMO), and the GLI transcription factors [2]. Normal activation of the hedgehog signaling pathway regulates multiple aspects of animal development, tissue homeostasis, regeneration [3], stem cell maintenance and tissue homeostasis [4]. Abnormal activation of the Hh pathway has been shown to contribute to tumorigenesis, progression, metastasis, and drug resistance in various cancers, including basal cell carcinoma (BCC) [5], medulloblastoma (MB) [6], and many other solid and hematological tumors [4]. In addition, the abnormal activation of Hh signaling has also been linked to the pathologies of breast [7], lung [8], pancreas [9], and prostate cancers [10, 11]. Nowadays, with the deepening of research on it, the abnormal activation mechanism of HH signaling pathway is divided into four categories: ligand-independent signaling, ligand-dependent autocrine signaling [12], ligand-dependent Hh signaling in a paracrine or reverse paracrine manner [13]. To understand the effects of these four types of abnormal activation mechanisms on tumorigenesis and development and the differences between them is very important for the study of tumorigenesis mechanisms and treatment strategies. This review focuses on the abnormal activation mechanism of the Hh pathway and its related tumors.

## Mechanism of Hedgehog signaling pathway

Essential in controlling development [14], tissue homeostasis [15], and regeneration [3, 16, 17], the Hedgehog (Hh) signaling system comprises ligands, receptors, and transcription factors.The secreted signaling proteins known as Sonic hedgehog (Shh), Indian hedgehog (Ihh), and Desert hedgehog (Dhh) have evolved from their original form in fruit flies, which only have one Hh ligand, into three homologs in mammals [18]. It has recently been discovered that the Smoothened (Smo) extracellular domain (ECD) mutation is critical for controlling the location of Smo cilia and high-level Hedgehog (Hh) signaling [19]. This mutation was previously believed to be non-essential in vertebrates. It is crucial to target Gli transcription factors for possible cancer therapeutics because multiple studies have shown that their deregulation in stem or progenitor cells can initiate carcinogenesis [20]. The work conducted by Alexandre et al. shown that Ci acts as a transcriptional activator in the Hh pathway, since elevated Ci levels can activate the patched gene (ptc) and other Hh target genes even in the absence of Hh activity [21].

Furthermore, co-culture experiments with Ptch1 overexpression and Ptch1 small interfering RNA (siRNA) transfected cells revealed that Ptch1 can exert non-cell autonomous inhibition on Smoothened (Smo) [22]. The transcriptional activation of the PTCH1 gene within the Hh-signaling pathway relies on a single functional Gli-binding site [23]. The Shh-Ptch1-Gli1 signaling pathway is implicated in the development and progression of colorectal tumors [24]. Gli proteins (GLI1, Gli2, and GLI3) bind in the PTCH1 promoter region to increase transcription. Strong suppression of both baseline and induced PTCH1 transcription results also from reduced GLI3 expression. As so, a single functional binding site for Gli1 determines the transcriptional activation of the PTCH1 gene mediated by the Hh signaling pathway [24].

**Fig. 1:**
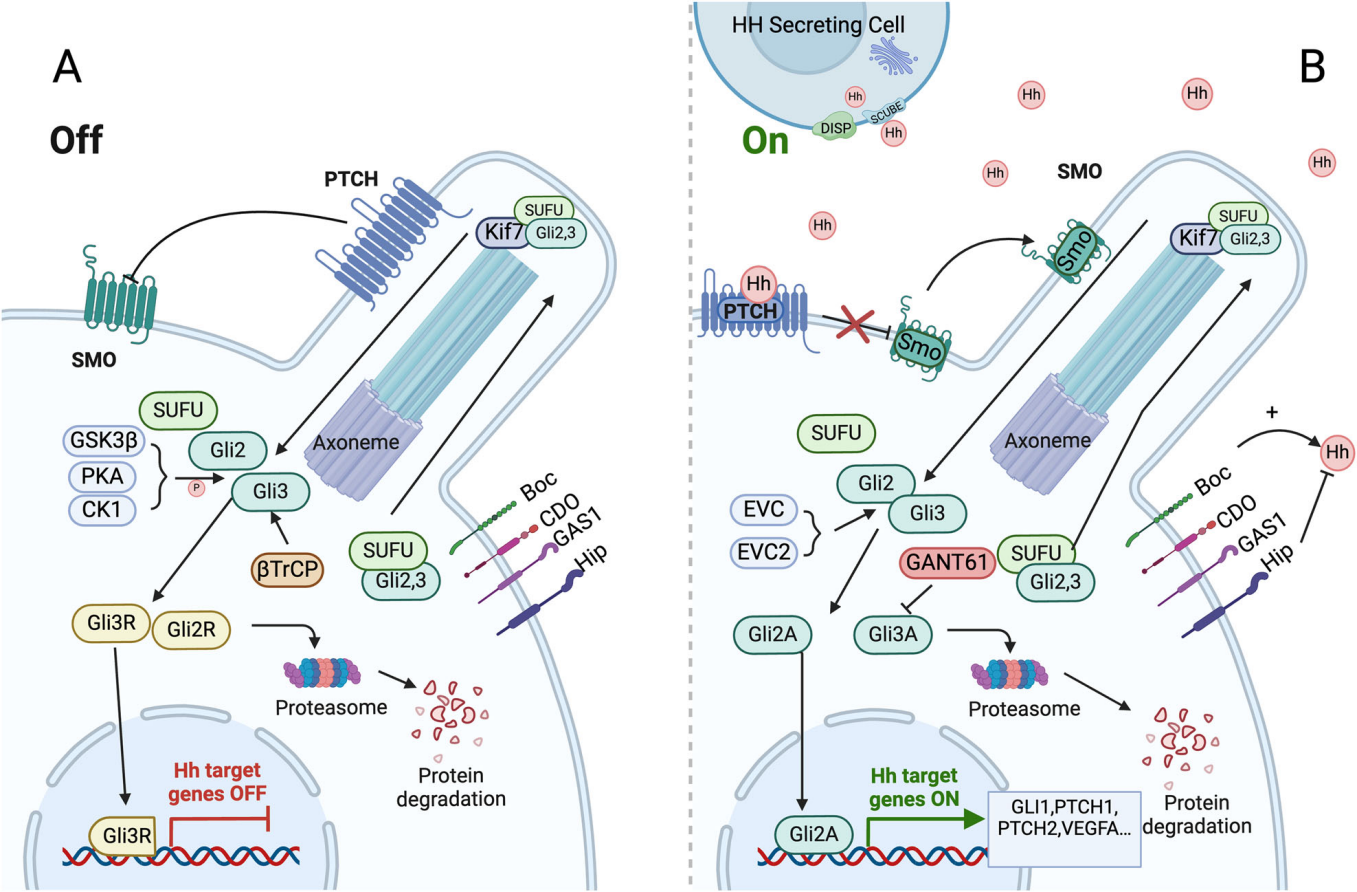
Normal activation mechanism of hedgehog signaling pathway. **A** Without HH ligand activation: Ptch1 inhibits Smo, preventing it from translocating to the cilium. SuFu binds with Gli2/3 and moves into the cilium, where Gli2/3 is phosphorylated by CK1, PKA, and GSK3β, causing Gli2/3 to separate from SuFu. Gli2 and Gli3 then cross the nuclear membrane into the nucleus, where they repress the transcription of target genes, with Gli3 being the primary repressor. **B** With HH ligand activation: The HH secreting cell releases the HH ligand, activating Smo and causing it to translocate to the cilium. SuFu binds with Gli2/3 and moves into the cilium, where Gli2/3 is phosphorylated by EVC and EVC2, causing Gli2/3 to separate from SuFu. Gli3 remains in the cytoplasm, while Gli2 crosses the nuclear membrane into the nucleus, where it initiates the transcription of target genes. Created with BioRender.com.

This research revealed that the Hh signaling pathway activates its cascade by inhibiting the secretion of 3β-hydroxysteroid (pro-vitamin D3), which depends on Ptch1. This inhibition releases the inhibitory effects on Smoothened (Smo) and the downstream transcription factor Gli. This finding not only clarifies the contradictory cause of Smith-Lemli-Opitz syndrome (SLOS) but also confirms that Hh acts as a unique morphogen. Its binding to one cell can activate Hh-dependent signaling cascades in other cells [22]. Together, these studies highlight the complex and important role of Hh signaling pathways in cell localization, gene regulation, and disease mechanisms (Fig. 1).

## Hedgehog signaling pathway in cancer

### Ligands of Hedgehog signaling pathway in cancer

Sonic Hedgehog (SHH) is one of the three Hedgehog (Hh) ligands and has been extensively studied for its crucial role in central nervous system (CNS) development. It influences cell fate determination, pattern formation, axon guidance, and the survival, proliferation, and differentiation of neurons [25]. Therefore, many neurological disorders are linked to disruptions in SHH signaling pathways. Beyond its role in development and neurological health, Shh also plays a important role to the occurrence and development of cancer, such as lung [26],prostate [27], breast [28], colon [29], ovarian [30], pancreatic [31], hepatocellular carcinoma [32], bladder [33] and renal cell carcinoma [34]. In renal cell carcinoma(RCC), Shh signaling plays a role in the progression of it, along with epithelial-mesenchymal transition (EMT) [34]. Dormoy et al. through two experimental methods, quantitative RT-PCR and immunoblotting, observed that in RCC, even in most cases, the von Hippel-Lindau (VHL) tumor suppressor gene was inactivated, but SHH signaling pathway remained activated [35]., this indicates the universality of the SHH signaling pathway in RCC. Moreover, TGF-β1 can induce Shh signaling, which in turn enhances bladder cancer cell migration, clonogenicity, and invasiveness by promoting EMT and bladder cancer stemness [33]. Nedjadi et al. further reported that high Shh expression is linked to lymph node metastasis in bladder cancer [36], emphasizing the significance of SHH signaling in cancer dissemination. And in breast cancer, Shh overexpression is a key event, with Shh promoter hypomethylation and NF-κB upregulation responsible for the observed increase in Shh expression [28]. In summary, the SHH signaling pathway is not only essential for CNS development but also plays a universal role in the progression of various cancers.

The Indian Hedgehog (IHH) gene, expressed in prehypertrophic and hypertrophic chondrocytes, is crucial for endochondral ossification, regulating chondrocyte differentiation, promoting proliferation and modulating osteoblast function [37]. Moreover, IHH has also been implicated in the development of cancer, including pancreatic cancer [38], colorectal cancer [39], and invasive ductal carcinoma of the breast [40]. In pancreatic cancer, the expression of Ihh and its receptor is closely related to the development of cancer. Relevant research shows that inhibiting the Hedgehog signaling pathway significantly inhibited the growth of pancreatic cancer cells [38], suggesting that abnormal activation of Ihh signaling is involved in cancer progression.

Desert Hedgehog is a key signaling molecule that influences gonadal development and plays an important role in testicular development, particularly during the differentiation of testicular mesenchymal stem cells into testosterone-secreting stromal cells [41, 42]. In addition to affecting gonadal development, DHH is also associated with the development of a variety of cancers. Proliferation of mouse glioblastoma stem cells is associated with inhibition of DHH-induced gamma-glutamyl cyclotransferase knockdown [43], while plexiform neurofibromatosis is associated with loss of Nf1 in DHH-expressing cells [44]. This suggests that DHH plays an important role in the reproductive system and the development of some tumors, as well as drug interventions targeting DHH signaling pathways, may become potential strategies for the treatment of cancer.

### Receptor of Hedgehog signaling pathway in cancer

The Patched (Ptch) protein receptor plays an important role in the regulation of the Hedgehog signaling pathway. It exists in two forms, Ptch1 and Ptch2, both of which serve as primary binding sites for the Sonic hedgehog (Shh) ligand [45]. Additionally, the transmembrane protein Smoothened (Smo) is a key component of this pathway. Normally, Patched inhibits Smoothened, preventing the activation of the Hh signaling; however, in the absence of Patched, Smoothened becomes structurally activated, which can lead to tumor development [46].

In the Hh signaling pathway, Ptch1 is regard as the primary regulator, but the concurrent loss of both Ptch1 and Ptch2 results in more severe tumorigenesis than the loss of Ptch1 alone [47, 48], highlighting the cooperative role of these two proteins in tumor suppression. However, their expression patterns are not completely overlapping. PTCH2, which is mainly expressed in germ cells, is located on chromosome 1p33-34. This chromosomal region is often deleted in certain reproductive cell tumors, indicating that PTCH2 may act as a tumor suppressor [49]. Furthermore, Ptch1 influences the intracellular positioning of cyclin B1, linking its tumor-suppressive function to the regulation of cell division [8]. This function is particularly critical in tumor-associated precursor cells found in nevoid basal cell carcinoma syndrome (NBCCS) [50]. In summary, Ptch1 and Ptch2 may play an important role in tumor inhibition, and their functions overlap but are unique. These findings provide valuable information for future cancer research and treatment.

### Transcription factors of Hedgehog signaling pathway in cancer

The Gli family of transcription factors, comprising Gli1, Gli2, and Gli3, plays distinct roles in the Hedgehog (Hh) signaling pathway. Gli1 primarily functions as a transcriptional activator of downstream target genes. Gli2 and Gli3 have the opposite effect, Gli2 mainly activates gene transcription, while Gli3 mainly acts as a repressor. A critical difference between Gli1 and Gli2 is Gli1’s capability to counteract the repressive function of Gli3 [51]. Gli3 repressor formation and activation of Gli1 and Gli2 combine to act on the cellular response to Hedgehog signaling [52, 53].

The dysregulation of the Hedgehog (Hh)-Gli signaling pathway is increasingly recognized as a key factor in the development of various human cancers, including basal cell carcinoma (BCC) [54], medulloblastoma (MB) [55], and embryonal rhabdomyosarcoma (eRMS) [56, 57], which are the three primary tumors associated with Gorlin syndrome [58]. Additionally, Gli involvement has also been observed in non-small cell lung cancer and many primitive neuroectodermal tumors [59]. In Gli-related cancers, cell proliferation is enhanced through the Gli-dependent expression of cyclin D1/D2 or N-myc proto-oncogenes [60]. But the Gli family does not always act together on cancer cells, for example, nearly all BCCs express Gli1, but not Gli3 or SHH, suggesting that Gli1, which can be induced by SHH, may serve as the principal oncogenic agent [61].

### Crosstalk between Hedgehog signaling pathway and other signaling pathways in cancer

There is substantial evidence of crosstalk between the Hedgehog (Hh) signaling pathway and other key pathways in various tumor types [62]. This interaction is particularly crucial in the resistance of cancer stem cells (CSCs) to treatment [63]. Notably, both Notch and HH pathways are concurrently activated in desmoid tumors and mesenchymal cell lines derived from desmoid tumors [64]. Meanwhile, When Patched, a negative regulator of Hedgehog, is disrupted in mice, it leads to the development of medulloblastoma with enhanced Notch signaling [65]. However, a mutually exclusive relationship between the Hedgehog and Notch pathways has been observed in skin cancer [5]. This complex interaction may depend on the specific tumor microenvironment. In docetaxel-resistant prostate cancer cells, there is a notable lack of differentiation markers accompanied by upregulation of both the Notch and Hedgehog pathways [66]. Notch may inhibit Hedgehog activity by suppressing Gli1 transcription through Hes1. Targeting both pathways concurrently may offer a more effective strategy for tumors cells [67].

Overexpression of Gli1 inhibits Wnt pathway activity, leading to reduced nuclear β-catenin accumulation and decreased proliferation of AGS cells [68]. This crosstalk between the Hh and Wnt pathways presents potential therapeutic opportunities for treating gastric cancer. Notably, overexpression of ZnRF3 not only inhibits Lgr5, a critical component of the Wnt pathway, but also significantly reduces Gli1 expression, a key transcription factor in the Hh pathway. These findings suggest that ZnRF3 suppresses the proliferation of gastric cancer cells and induces apoptosis by down-regulating both the Wnt and Hh pathways [69]. Such crosstalk has also been identified as a crucial factor in the recurrence, invasion, and metastasis of colon cancer [70].

Moreover, The RAS signaling pathway is intricately linked with the Hedgehog (Hh) pathway in promoting cell proliferation and survival, particularly in melanoma. This interaction is facilitated by the regulation of Gli1’s nuclear localization and transcriptional activity, which is crucial for cancer development [71]. In addition, research has shown that activation of the RAS/MAPK pathway (KRAS), through various upstream signals and converging at Gli transcription factors, plays an important role in the development of pancreatic tumors [72]. In summary, crosstalk between the Hedgehog signaling pathway and the RAS signaling pathway can affect different tumors.

### Abnormal activation of Hedgehog signaling pathway and associated cancers

The abnormal activation of the Hedgehog (Hh) signaling pathway not only accelerates the proliferation of cancer cells but also maintains the population of cancer stem cells and cancer-associated fibroblasts (CAFs) across a range of cancers, including lung cancer [8]. As research progresses, the mechanisms of abnormal Hedgehog (Hh) signaling pathway activation are now categorized into four types: ligand-independent signaling, ligand-dependent autocrine signaling, ligand-dependent Hh signaling in a paracrine or reverse paracrine manner [12, 13] (Table 1).

**Table 1:** The occurrence of different mechanisms in various cancer types.

Classification of cancer	Cancer	Abnormal activation type	Cause of pathway activation	Reference
Skin system	BCC	Ligand-independent autocrine patterns	Dual targeting of Ptch1 and Ptch2, as well as overexpression of key Hh signaling mediators.	[48]
Nervous system	MB	Ligand-independent autocrine patterns	Mutations in the PTCH1, SMO and SUFU	[84]
Soft tissue sarcoma	RMS	Ligand-independent autocrine patterns	Partial inactivation of PTCH1	[78]
Digestive system	CRC	Ligand-dependent autocrine patterns	Overexpression of Hh/Gli components	[101–103]
		Ligand-dependent paracrine patterns	Activation of surrounding stromal cells promotes tumor cell growth, changes tumor microenvironment and enhances tumor metastasis.	[135]
	Pancreatic cancer	Ligand-dependent autocrine patterns	TNF-α, IL-1β and hypoxia-induced upregulation of Smo	[110]
		Ligand-dependent paracrine patterns	HH ligands enhance tumor growth, invasion, metastasis and perineural invasion through paracrine mechanisms.	[133]
Urogenital system	Bladder cancer	Ligand-dependent autocrine patterns	Shh-expressing stem cells in the basal urothelium activate the HH pathway.	[115]
	Prostate cancer	Ligand-dependent autocrine patterns	Increased Hh ligands (especially Ihh and Dhh) and changes in reactive substrates (such as decreased SMCs) in the tumor microenvironment.	[121]
		Ligand-dependent paracrine patterns	By promoting osteoblast development and SEMA3C induces androgen production, it affects tumor progression and metastasis.	[138–140]
	Breast cancer	Ligand-dependent autocrine patterns	Up-regulated stem cell markers such as OCT4, NESTIN and NANOG	[125]

Table 1 lists the various forms of cancer in the digestive, urogenital, skin, nervous, and soft tissue sarcoma systems, along with the abnormal activation types of the Hh signaling pathways that correspond to them (such as ligand-independent autocrine mode and ligand-dependent autocrine/paracrine mode). It also describes the precise causes of abnormal activation of these pathways. such include alterations in the cell microenvironment, protein overexpression, and gene mutation. Furthermore, these findings offer potential targets for upcoming treatment approaches in addition to crucial insights into the part Hh signaling pathways play in the genesis of cancer.

### Hh signaling of autonomous and ligand-independent types

**Fig. 2:**
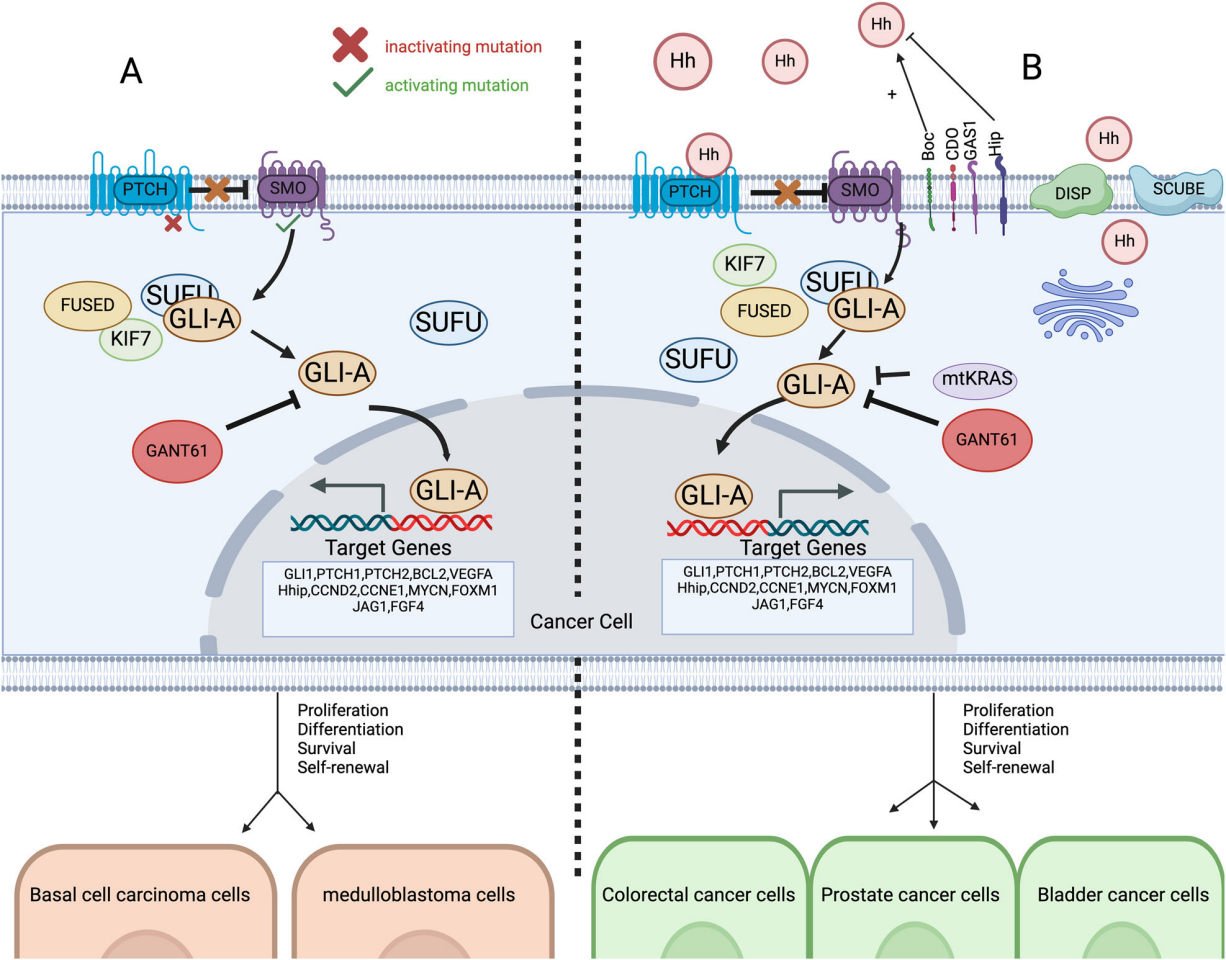
Autocrine of the hedgehog signaling pathway. **A** Ligand-Independent Activation: In this scenario, the HH pathway is activated without the presence of the HH ligand. The Patched (Ptch) receptor is inactive, allowing the Smoothened (Smo) protein to remain active. This leads to the activation of the Gli-A transcription factor, which then moves into the nucleus and promotes the transcription of target genes that drive cancer cell proliferation. **B** Ligand-Dependent Activation: Here, the HH pathway activation depends on the presence of the HH ligand. The HH ligand binds to the Ptch receptor, which results in the inhibition of Ptch activity. This inhibition allows Smo to activate, leading to the activation of the Gli-A transcription factor. Gli-A then translocates to the nucleus and induces the expression of target genes involved in cancer progression. Created with BioRender.com.

Recent studies have shown that during both development and tumorigenesis, the ligand-independent autocrine hedgehog signaling pathway can induce the expression of Gli1 independent of Hh/Smo signaling [73, 74]. This abnormal activation mechanism influences the development of a variety of tumors, such as basal cell carcinoma [75], medulloblastoma [76], meningiomas [77] and Rhabdomyosarcoma [78] (Fig. 2).

### Neoplasms of skin system

Ptch1 and Ptch2, both crucial in suppressing tumor growth in basal cell carcinoma (BCC) cells. However, when both are targeted at the same time, they can unexpectedly activate the Hedgehog (Hh) signaling pathway without bounding ligand, which may act promote tumor growth [48]. Additional evidence for the critical role of activated Hh signaling in BCC development has been obtained from genetic mouse models and skin grafting experiments. Various methods have demonstrated the involvement of Hh signaling in BCC formation. Heterozygous ptc + /7 mice, when exposed to UV irradiation, develop features similar to BCC, although spontaneous BCC formation is rare in mice [75]. Additionly, grafting human keratinocytes expressing Sonic hedgehog (SHH) onto the backs of nude mice results in the formation of BCC-like structures [79]. Moreover, overexpression of key Hh-signaling mediators, including SHH, GLI1, GLI2, and an oncogenic form of SMOH, in the epidermal cells of transgenic mice, leads to the induction of BCC-like tumors [80]. The above conclusions can prove that abnormal activation of HH signaling pathway plays an important role in the development of BCC.

### Nervous system neoplasms

Medulloblastoma (MB), one of the most prevalent malignant brain tumors in children, originate from various distinct populations of neural stem cells or progenitor cells during early development [81]. It is categorized based on molecular and histological characteristics into WNT-activated, SHH-activated TP53 wild type, SHH-activated TP53 mutant, and non-WNT/non-SHH subgroups, with the ‘SHH-activated’ group being driven by the activation of the Hedgehog pathway, often associated with desmoplastic histology [76, 82]. This group arises due to mutations in PTCH1, SMO, and SUFU, or through the amplification of GLI1, GLI2, CCND2, and N-MYC8. Among them, PTCH1 mutations is the most frequent drivers of MB [83–87]. Common cytogenetic events in this subgroup include the loss of chromosome 9q, which results in the loss of heterozygosity of PTCH1, and the loss of chromosome 10q, leading to the loss of SUFU [88]. Moreover, SHH-MB frequently exhibits recurring changes in the copy numbers of genes involved in the p53 pathway. Disruption of p53 signaling can result in issues with cell-cycle regulation, apoptosis, and DNA repair [86]. The Hh pathway maintains cancer stem cell (CSC) characteristics in MB through regulators like Nanog, which is involved in the GLI gene family, and governs the self-renewal and proliferation of cancer stem cells by downstream mediators such as NFκB [89].

### Soft tissue sarcoma

In Rhabdomyosarcoma (RMS), the Hedgehog (Hh) signaling pathway is critically involved in the occurrence and development of tumors. This involvement was first identified by Hahn et al. in 1998, who discovered that mice with partial inactivation of PTCH1 exhibited a higher incidence of embryonal RMS (ERMS), a specific RMS subtype [90]. This early work suggested that the Hh pathway might be a therapeutic target in RMS. Further research by Almazán-Moga et al. demonstrated that down-regulation of IHH, DHH, and GLI1 significantly reduced the expression of GLI1, GLI2, and PTCH1. Notably, suppression of SHH did not affect GLI1 levels but significantly lowered GLI2 and PTCH1 expression [78], indicating that while SHH is present in a minor fraction of RMS cell lines and tumors, IHH and DHH are the primary Hh ligands in RMS. This research highlights the complexity of Hh signaling in tumorigenesis and implies that targeted therapies against these ligands may be more effective than those targeting SHH.

### Ligand-dependent carcinogenic Hh signaling in autocrine mode

Multiple studies have shown that the ligand-dependent autocrine Hedgehog signaling pathway is overexpressed in a range of tumors, such as those affecting the stomach [91], esophagus [92], pancreas [13], colon [93], ovaries [94], uterus [95], breasts [96], prostate [97], lungs [98], Bladder Cancer [99] and gliomas [100].

### Digestive system neoplasm

The Hedgehog (Hh) signaling pathway plays a dual role in the colonic epithelium, promoting Paneth cell differentiation and regulating the development of colonic epithelial cells through autocrine signals [96]. However, in colorectal cancer, the Hh pathway is persistently activated through ligand-dependent mechanisms, which can involve both canonical and non-canonical pathways. This activation often leads to overexpression of Hedgehog/Gli components, including Shh, PTCH1, SMO, and Gli [101–103]. Epigallocatechin gallate (EGCG) has been shown to inhibit colon tumor growth by targeting the Shh and PI3K pathways, inducing apoptosis, and reducing cancer cell migration and invasion. Thus, EGCG holds potential as a chemotherapeutic agent for colorectal cancer [104], suggesting its potential as a chemotherapeutic agent

Additionally, research by Huang et al. indicates that lymphatic metastasis significantly contributes to colorectal cancer (CRC) progression, with lymphangiogenesis in CRC being regulated by pathways such as Sonic Hedgehog (Shh) signaling [105]. The expression levels of Hh components may modulate the local immune response and epithelial barrier integrity in CRC [106]. Additionally, a variant of the Hedgehog signaling pathway, functioning independently of GLI, has been observed in cancer organoids rich in CSCs, potentially sustaining the undifferentiated state of these cells [107]. In vivo studies have confirmed the role of the Hedgehog-GLI (HH-GLI) pathway in preserving the self-renewal capacity of CSCs, including CD133+ colon CSCs [108]. These findings underscore the complexity of the Hh signaling pathway in colon cancer and suggest that targeted therapies against this pathway could be effective in treating CRC.

The Sonic Hedgehog (SHH) signaling system is a key factor in the development of pancreatic cancer, influencing the tumor microenvironment and encouraging the growth of cancer cells [109]. According to research by Wang et al., tumor necrosis factor alpha and interleukin-1 beta in stromal hyperplasia activate the SHH pathway, which involves both canonical and non-canonical processes, promoting the growth of pancreatic ductal adenocarcinoma [110]. The intricate relationship between the tumor and its surroundings is highlighted by this activation.

The epithelial-mesenchymal transition (EMT), a crucial step in the early phases of pancreatic carcinogenesis that promotes cell dispersion, is also connected to the activation of the Hedgehog signaling system [111]. This implies that the Hedgehog signaling pathway may be activated early in the development and spread of pancreatic cancer. According to research by Kimberly Walter et al., the Hedgehog receptor Smo was expressed more frequently in human pancreatic cancer-associated fibroblasts (CAFs) than in normal pancreatic fibroblasts. In CAFs, Smo activates Gli1 expression by sending Sonic Hedgehog signals. Gli1 activation in these cells was inhibited by Smo knockdown with short interfering RNA [112], indicating the potential of targeting Smo as a therapeutic approach to interfere with the pro-tumorigenic SHH signaling in the microenvironment of pancreatic cancer. Further more, Jeng et al. have shown that hypoxia can directly induce the Hh pathway in PDAC cells by upregulating the transcription of Smoothened (Smo), independent of ligand binding. This ligand-independent activation enhances the invasiveness of PDAC cells [113]. Therefore, abnormal activation of Hedgehog signaling pathway, both ligand-dependent and non-ligand-dependent, has been implicated in the development of pancreatic cancer and may be a potential target for its treatment.

### Urogenital neoplasms

Originating from different paths, bladder cancer can be categorized as non-muscle-invasive or muscle-invasive [114]. The Sonic Hedgehog (Shh) molecule suppresses Ptch during urothelial mesenchymal development, therefore activating the Gli transcription factor in target cells. With mega bladder mgb−/− mutant mice, DeSouza and colleagues examined Shh expression patterns in both normal and aberrant bladder development. This work revealed unique regional and temporal patterns of Shh signaling components across bladder development [115]. By modifying or eliminating particular cells, Shin et al. showed that muscle-invasive bladder carcinomas develop only from Shh-expressing stem cells in the basal urothelium [116]. This result offers a fresh viewpoint for explaining the source of invading bladder cancer. Furthermore linked to a range of cancer-promoted metabolic processes including enhanced glycolysis, nucleotide metabolism, and amino acid metabolism is raised activity of the Hedgehog signaling pathway in bladder cancer [117]. Changes in these metabolic pathways might give cancer cells the energy and biomacromolecules they need to proliferate and survive, therefore promoting cancer formation and spread.

Using Ptc1lacZ and Gli1lacZ reporter mice to trace Hedgehog (Hh) pathway activation, Bushman et al., studying activation of the Hedgehog (Hh) signaling pathway in the adult prostate, observed rather rare and scattered epithelial staining in the adult prostate, indicating that autocrine Hh pathway activation is limited to a small subset of epithelial cells, particularly during prostate development [118]. Although blocking Hh signaling can help to lower tumor invasion and metastases, long-term suppression may cause treatment resistance to develop [119]. Preclinical studies in prostate cancer also support this. Autocrine signaling often stimulates the route in prostate cancer [120], inting that blocking this signaling system may not impair normal prostate development but could improve the response to castration and impede tissue regeneration when testosterone is reintroduced [118, 121]. Using GANT61 or genistein, an isoflavone present in soybeans, inhibition of the Hh pathway essentially prevented tumor phere and colony development [122]. This finding further confirms that the Hh signaling pathway plays a key role in the maintenance of prostate cancer stem cells.

In breast cancer, Hedgehog signaling has been shown to promote early tumorigenesis by enhancing tumor cell proliferation [123] and play a vital role in the self-renewal and differentiation of breast cancer stem cells [124]. Studies related to breast cancer have shown that inhibition of Hh signaling in MCF-7-derived CD44+/CD24− CSCs led to a reduction in cell numbers, accompanied by downregulation of stem cell markers such as OCT4, NESTIN, and NANOG. This suggests that Hh signaling helps maintain a self-renewing profile in breast CSCs by upregulating these key stem cell markers [125].

### Ligand-dependent Hh signaling in paracrine or reverse paracrine mode

**Fig. 3:**
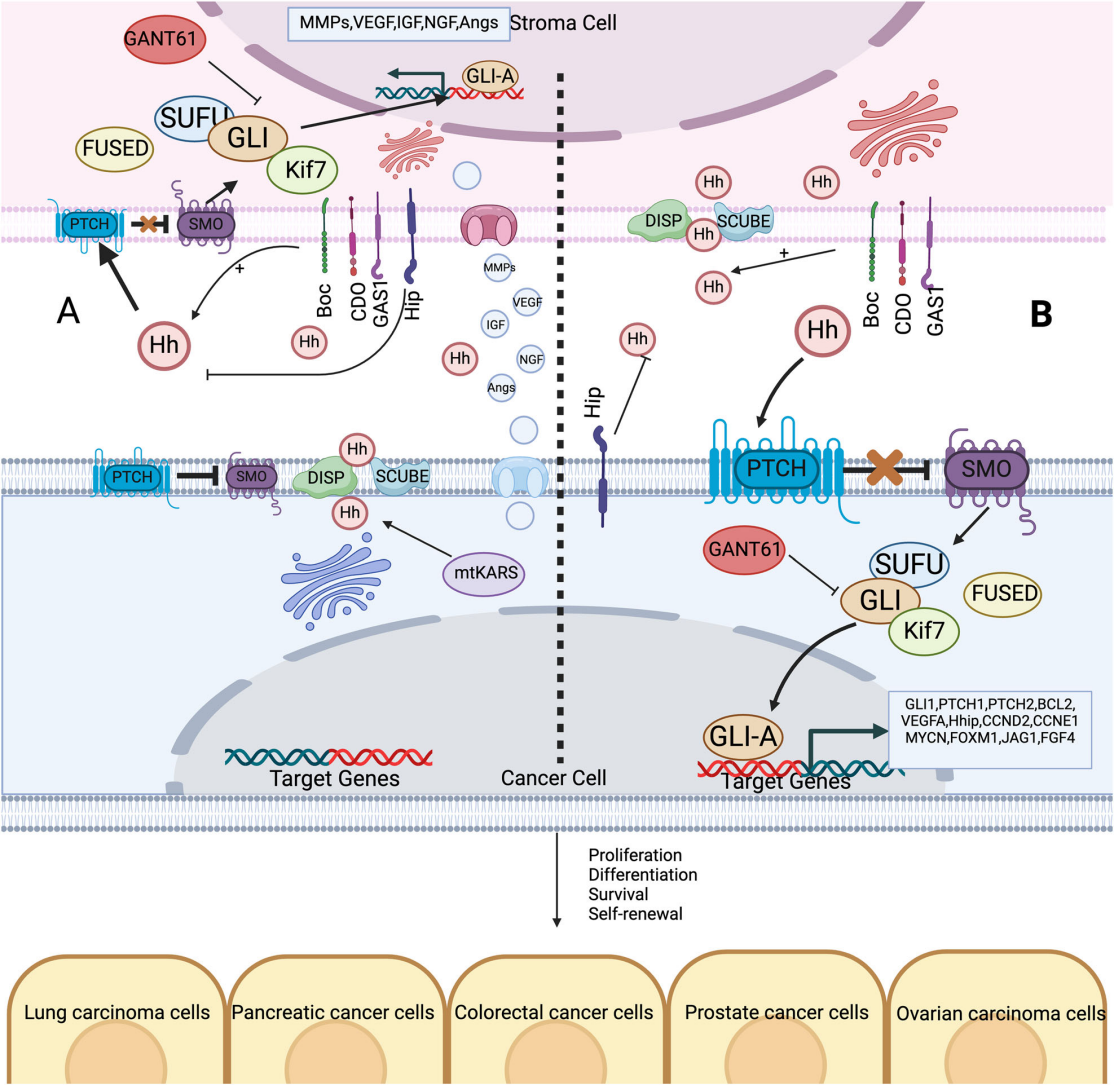
Paracrine of the hedgehog signaling pathway. **A** Paracrine Signaling: Stroma cells release HH ligands (SHH, IHH, DHH), which bind to the Ptch1 receptor on the cancer cell. This binding inhibits Ptch1, allowing Smo to become active. Active Smo leads to the activation of Gli transcription factors (Gli-A), which then translocate into the nucleus to promote the transcription of target genes. **B** Reverse Paracrine Signaling: Cancer cells release HH ligands (SHH, IHH, DHH), which bind to the Ptch1 receptor on the stroma cell. This binding inhibits Ptch1, allowing Smo to become active in the stroma cell. Active Smo then leads to the activation of Gli transcription factors (Gli-A), which then translocate into the nucleus to promote the transcription of target genes. Created with BioRender.com.

Paracrine Hedgehog (Hh) signaling plays a crucial role in the development and maintenance of various epithelial structures [126]. In epithelial cancers such as lung [127], prostate [128], colon [129], pancreatic [130], and ovarian [131] cancer without mutations in the Hh pathway, tumor-expressed Hh ligands stimulate tumor growth indirectly by activating Hh signaling in the surrounding stroma. This activation creates a microenvironment conducive to tumor progression [13] (Fig. 3).

### Digestive system neoplasm

Research on ligand-dependent paracrine signaling has revealed that pancreatic cancer development is much influenced by it. Using a tissue-specific gene activation model, Marina et al. shown how Hedgehog ligands contribute to tumor development [130]. Furthermore supporting paracrine signaling in pancreatic ductal adenocarcinoma (PDA), in a Pdx1-Cre, LsL-KrasG12D, and Ink4a/Arflox/lox transgenic mouse model [132]. Hedgehog ligands Using a Ptc-LacZ reporter mouse, Hua et al. demonstrated via paracrine processes that tumor-derived Hh ligands induce PDA [133]. These results imply that Hh ligands not only function inside tumor cells but also influence the surrounding cells and microenvironment, hence promoting tumor growth. In animal studies, co-implantation of paracrine Shh-activated cells also enhances tumor cell invasion in the trunk, triggers nerve dysfunction, and promotes orthotopic xenograft tumor development, metastases, and perineural invasion [134]. These findings underline the several functions of paracrine Shh signaling in pancreatic cancer progression: tumor behavior linked with neurological dysfunction, and tumor cell invasiveness and metastases promotion.

In colorectal cancer (CRC), tumor microenvironment is substantially influenced by elevation of Hh ligand expression. Two ways may be used to achieve this effect: paracrine support, in which cancer cells secreted by them subsequently act on surrounding cells, so influencing tumor development and invasion; or autocrine action, in which case Hh ligands act directly on the ligand-producing cancer cells themselves, so contradicting paracrine action and resulting with different biological effects [135, 136]. New understanding of the function of the Hh signaling pathway in CRC is offered by the work of Marco Gerling et al. Reduced activation of the Hh signaling system, they discovered, helps colorectal cancer linked with colitis in a mouse model develop. This implies that under some conditions the Hh signaling pathway might be inhibitory for intestinal inflammation and tumor formation. Nevertheless, the Hh signaling pathway was able to stop tumor formation when it was especially triggered in stromal cells of the tumor microenvironment [137]. This implies that the type of cell where the Hh signaling pathway activates determines the complicated function of the system in CRC.

### Urogenital neoplasms

Paracrine Sonic hedgehog (Shh) signaling drives osteoblast development in the bone microenvironment in metastatic settings, therefore enabling prostate cancer spread [138, 139]. Furthermore underlining the complex functions of paracrine signaling in prostate cancer progression is SEMA3C-induced androgen production in prostatic stromal cells [140]. Recent studies highlight the complex roles of paracrine Hedgehog signaling in both cancer and non-cancerous settings, therefore impacting different biological processes and tumor microenvironment. Paracrine Hh signaling affects epithelial ductal development in prostate cancer, presumably mediated by interactions of complex tissue microenvironment [128]. Moreover, Hh-driven steroidogenesis by stromal cells in prostate cancers may help tumor development and progression to a castration-resistant state [141].

### Hedgehog pathway inhibitor in cancer therapy

Inhibitors targeting the Hedgehog pathway have shown promising outcomes in clinical trials, with ongoing evaluations. Vismodegib (GDC-0449), an orally administered inhibitor of the Hedgehog signaling pathway targeting SMO protein, has progressed furthest in clinical development. Initial trials in basal cell carcinoma and medulloblastoma have demonstrated significant efficacy and safety [142]. In January 2012, vismodegib became the first FDA-approved drug targeting the Hedgehog (Hh) pathway, based on favorable results from phase I and II trials showing its effectiveness against basal cell carcinoma (BCC) [143]. GDC-0449 is a low molecular weight inhibitor of the tumor-promoting hedgehog (Hh) signaling pathway, and the ability of GDC-0449 and related compounds to inhibit two key ABC transporters may contribute to its effectiveness in treating malignant tumors [144]. Sonidegib, as an smo inhibitor, has shown sustained efficacy and a manageable safety profile in the treatment of patients with advanced basal cell carcinoma [145]. This approval marks a significant advance for Hh signaling pathway inhibitors in the treatment of basal cell carcinoma and provides a new therapeutic option for other potential indications.

Inhibitors that target the Hedgehog (Hh) signaling pathway have shown potential in clinical trials to inhibit tumor growth and metastasis. To be specific, Arsenic compounds like sodium arsenite, arsenic trioxide (ATO), and phenylarsine oxide (PAO) effectively inhibit the response of the Sonic hedgehog (Shh) amino-terminal domain (ShhN). Arsenic trioxide (ATO) specifically targets GLI1 levels by binding to the GLI1 protein, thereby suppressing its transcriptional activity and reducing expression of endogenous GLI target genes. This mechanism leads to significant inhibition of human cancer cell growth and tumor development in animal models [146, 147]. Moreover, Cyclopamine, another inhibitor, disrupts Hedgehog signaling in vertebrate animals by binding to the seven-helical receptor of the Smoothened (Smo) protein. In vitro studies demonstrate that cyclopamine inhibits cell proliferation and alters gene expression patterns associated with neuronal differentiation, showing promise in preclinical models of medulloblastoma [148, 149].

Cancer stem cell self-renewal is under control by the SHH/SMO/GLI signaling system. Targeting these cancer stem cells efficiently requires combining SHH signaling inhibitors with chemotherapy, radiation treatment, or immunotherapy [150]. Inoscavin A causes death dependent on Smo, the central Hedgehog pathway receptor. On the other hand, upregulating Smo expression reduces Inoscavin A’s dead effects on cells [151].

Garcinone C modulates non-canonical Hedgehog signaling pathways involving Gli1 to show effectiveness in preventing colon tumor development [152]. Based on the Sonic hedgehog (Shh) binding ring (HHIP), Owens et al. developed a cyclic peptide and carried out several rounds of affinity maturation screening for big cyclic peptide libraries generated in E. coli cells. We obtained an optimal macrocyclic peptide inhibitor (HL2-m5) using this approach which essentially inhibits SH-mediated hedgehog signaling pathway and GliL-regulated gene transcription in living cells [153]. Lea et al. detailed how cell screening helped to identify HH-pathway modulator Pipinib. Pipinib specifically inhibits phosphatidylinositol 4-kinase IIIβ (PI4KB) and reduces Glib-mediated transcription and Hh target gene expression by means of SMO translocation to cilia [154]. Thus, another route to limit SMO activity and Hedgehog signaling could be blocking PI4KB, so lowering phosphatidyl4-phosphate levels. Targeting glioma-associated oncogene homologous protein (gli), GANT61 is the first and most often utilized inhibitor of Hedgehog (Hh) signaling pathway [155]. Novel HH inhibitor with considerable potential in the treatment of hematological malignancies HH78 competitively binds to SMO and suppresses GLI transcriptional activity [156]. These investigations taken together expose a set of molecules that block the Hh signaling pathway via several channels. These inhibitors might become a major part of cancer treatment since a better knowledge of the function of the Hh signaling system in tumor formation helps to guide treatment (Table 2).

**Table 2:** The components involved for each type of signaling with available inhibitors and their current state in clinical trials.

Signal path element	Inhibitor	Mode of action	Clinical trial status	Reference
Smo inhibitors	Vismodegib	Keeped the transcription factors GLI1/2 in an inactive state.	Phase I and II trials	[142–144]
	sonidegib	Blocked the abnormal activation of the HH signaling pathway.	Phase I and II trials	[145]
	Cyclopamine	Binded to the seven-helix receptor of Smo protein.	Preclinical	[149]
	Inoscavin A	Reduced the expression levels of key proteins in the Hh signaling pathway, such as Shh, Ptch1, Smo and Gli1.	Preclinical	[151]
Gli inhibitors	Garcinone C	Inhibited AKT phosphorylation and induced G0/G1 arrest.	Preclinical	[152]
	GANT61	Its hydrolysate, GANT61-D, binded to a specific region of Gli1 protein.	Preclinical	[155]
	HH78	competitively binded to SMO and suppressed GLI transcriptional activity.	Preclinical	[156]
	ATO	Directly inhibited the transcriptional activity of GLI1 protein and blocked HH/GLI signaling pathway	Phase I, II and III trials	[146]
PI4KB inhibitors	Pipinib	Selective inhibition of PI4KB reduced PI4P levels and prevented HH-mediated ciliary localization of Smo protein.	Preclinical	[154]

Smo inhibitors (e.g., Vismodegib, Sonidegib, Cyclopamine, Inoscavin A), Gli inhibitors (e.g., Garcinone C, GANT61, HH78, ATO), and PI4KB inhibitors (e.g., Pipinib) are among the inhibitors listed in Table 2 that target distinct elements of the Hh signaling pathway. These inhibitors function in various ways. The clinical trial status of these inhibitors, including those undergoing phase I/II clinical trials and those still undergoing pre-clinical research, is also listed in Table 2. These findings offer a scientific foundation for upcoming medication development and clinical care, as well as a valuable resource for comprehending the mechanism of action and potential clinical uses of Hh signaling pathway inhibitors.Hedgehog signaling pathway inhibitor in the clinical trials from Public Chemical Database (https://pubchem.ncbi.nlm.nih.gov/).

## Conclusion and future directions

During development, the Hedgehog (Hh) signaling pathway is essential for controlling tissue patterning, cell differentiation, and proliferation. The pathophysiology of many cancers is primarily linked to dysregulation of the Hh signaling system. A thorough grasp of the subtleties of Hh pathway activation, including both autocrine and paracrine pathways, is essential for the effectiveness of targeted cancer therapies.Current therapeutic approaches, such as the use of Smoothened (Smo) inhibitors, have shown promise in the face of obstacles such drug resistance. Identifying new therapeutic targets within the Hh pathway and clarifying the interactions between Hh signaling and other cellular signaling pathways should be the main goals of future research projects. Advances in this field could greatly increase the effectiveness of cancer treatment plans and provide patients suffering from Hh pathway-associated tumors fresh hope.
